# Applying interpretable machine learning algorithms to predict risk factors for permanent stoma in patients after TME

**DOI:** 10.3389/fsurg.2023.1125875

**Published:** 2023-03-24

**Authors:** Yuan Liu, Songyun Zhao, Wenyi Du, Zhiqiang Tian, Hao Chi, Cheng Chao, Wei Shen

**Affiliations:** ^1^ Department of General Surgery, Wuxi People's Hospital Affiliated to Nanjing Medical University, Wuxi, China; ^2^ Department of Neurosurgery, Wuxi People's Hospital Affiliated to Nanjing Medical University, Wuxi, China; ^3^ Clinical Medical College, Southwest Medical University, Luzhou, China

**Keywords:** rectal cancer, permanent stoma, prognosis, risk factor, machine learning, surgery

## Abstract

**Objective:**

The purpose of this study was to develop a machine learning model to identify preoperative and intraoperative high-risk factors and to predict the occurrence of permanent stoma in patients after total mesorectal excision (TME).

**Methods:**

A total of 1,163 patients with rectal cancer were included in the study, including 142 patients with permanent stoma. We collected 24 characteristic variables, including patient demographic characteristics, basic medical history, preoperative examination characteristics, type of surgery, and intraoperative information. Four machine learning algorithms including extreme gradient boosting (XGBoost), random forest (RF), support vector machine (SVM) and k-nearest neighbor algorithm (KNN) were applied to construct the model and evaluate the model using k-fold cross validation method, ROC curve, calibration curve, decision curve analysis (DCA) and external validation.

**Results:**

The XGBoost algorithm showed the best performance among the four prediction models. The ROC curve results showed that XGBoost had a high predictive accuracy with an AUC value of 0.987 in the training set and 0.963 in the validation set. The k-fold cross-validation method was used for internal validation, and the XGBoost model was stable. The calibration curves showed high predictive power of the XGBoost model. DCA curves showed higher benefit rates for patients who received interventional treatment under the XGBoost model. The AUC value for the external validation set was 0.89, indicating that the XGBoost prediction model has good extrapolation.

**Conclusion:**

The prediction model for permanent stoma in patients with rectal cancer derived from the XGBoost machine learning algorithm in this study has high prediction accuracy and clinical utility.

## 1. Introduction

Rectal cancer is a gastrointestinal tumor with an extremely high morbidity and mortality rate. The incidence of colorectal cancer is increasing year by year due to changes in people's lifestyle and dietary habits. A 2019 epidemiological survey (1) showed that the incidence of colorectal cancer ranks third among malignant tumors worldwide, after lung cancer and breast cancer. Total mesorectal excision (TME), a common surgical treatment for rectal cancer, has greatly improved the prognosis of patients with rectal cancer. The principle of surgery is to completely resect the entire mesentery of low and intermediate rectal cancer to reduce the postoperative recurrence rate of patients while increasing the rate of anal preservation and improving the survival quality of patients (2–4). However, as the level of surgery continues to evolve, clinicians are gradually discovering the limitations of TME, such as patients’ vulnerability to serious complications such as anastomotic leakage after surgery (5, 6). The quality of life for patients who develop postoperative complications is poor and there is an increased risk of secondary surgery. Therefore, temporary prophylactic stoma is often used clinically for patients with rectal cancer who have preserved anus, thus reducing the pressure at the anastomosis and reducing the risk of anastomotic leakage (7). Fortunately, some patients can undergo ostomy reversal at the appropriate time to improve quality of life. However, other patients are unable to retract for various reasons and suffer great physical and psychological damage. Some studies (8, 9) have shown that permanent stoma prolongs the life of patients but reduces their quality of life. Therefore, it is crucial to understand high risk factors for permanent stoma so that surgeons can appropriate counsel patients.

Artificial intelligence (AI) is developing rapidly in the medical field (10). Machine learning, as a major branch of AI, has the advantages of more stable model building and more accurate prediction, is favored by clinicians and is used in clinical prediction and other aspects (11, 12). In this study, we analyzed the clinical information of rectal cancer patients and applied machine learning algorithms to establish a prediction model for permanent stoma in rectal cancer patients to aid clinicians in making timely and accurate individualized treatment plans.

## 2. Materials and methods

### 2.1. Study subjects

In the study, we used data from the clinical databases of Wuxi People's Hospital affiliated to Nanjing Medical University and Wuxi Second People's Hospital. Inclusion criteria: (1) patients were diagnosed with rectal cancer by pathological examination; (2) patients were treated with TME surgery; and (3) the surgical team consisted of senior doctors who had the ability to independently perform TME and enterostomy. Exclusion criteria were as follows: (1) patients with other malignant tumors; (2) patients who had been diagnosed with distant metastasis of rectal cancer; (3) patients with other diseases of the rectum; (4) patients diagnosed with life-threatening cardiovascular diseases such as cerebral infarction; (5) patients diagnosed with important organ diseases such as liver failure or kidney failure; and (6) missing case records or missed visits. Patients were followed up for at least three years after surgery. Two surgeons performed medical history, physical examination and abdominal ultrasound, computed tomography (CT) and other imaging examinations on patients every three months. The study was approved by the Ethics Review Committee of Wuxi People's Hospital, with approval number KY22086.

### 2.2. Study design and data collection

Clinical data were retrospectively collected from January 2010 to January 2018 from patients with rectal cancer at Wuxi People's Hospital and Wuxi Second People's Hospital, including 25 preoperative variables (within 24 h of the day of surgery) and intraoperative variables. Preoperative variables collected included patient demographic characteristics (gender, age, smoking history, alcohol history, and body mass index), basic clinical characteristics (American Society of Anesthesiologists score, nutrition risk screening 2002 score, history of surgery, chemotherapy, and radiotherapy), basic medical history (anemia, rectal stenosis, diabetes, hypertension, hyperlipidemia, and coronary artery disease), laboratory tests (carcinoembryonic antigen, carbohydrate antigen 19-9, and albumin), and tumor characteristics (T-category, N-category, tumor recurrence, tumor size, and tumor distance from the dentate line). Intraoperative variables collected included whether a permanent stoma was performed.

### 2.3. Definition of permanent stoma

A permanent stoma is defined as a permanent stoma created during the patient's initial surgery or a permanent stoma created during the progression of the patient's disease. With the patient's consent, the decision to create a permanent stoma is made by the surgeon, taking into account the patient's physical condition and disease progression.

### 2.4. Development and evaluation of predictive models for machine learning algorithms

SPSS software and R software were applied for the construction and evaluation of clinical prediction models. (1) Univariate and multivariate regression analyses were performed. The chi-square test was applied to categorical variables to compare the differences between the two groups; a *t*-test was performed for continuous variables that conformed to a normal distribution; and the rank sum test was selected for continuous variables that did not conform to a normal distribution. *P* < 0.05 indicated that the difference was statistically significant. Logistic regression analysis of variables with significance in the univariate analysis was performed to obtain independent influences on permanent stoma in patients with rectal cancer. (2) Evaluate and build prediction models. Rectal cancer patients from Wuxi People's Hospital from January 2010 to December 2016 were selected as the internal validation set, and rectal cancer patients from Wuxi Second People's Hospital from January 2017 to January 2018 were selected as the external validation set. The internal validation set was randomly divided into training set (70%) and test set (30%). The independent impact factors derived from the regression analysis were incorporated into four machine learning algorithm prediction models: support vector machine (SVM), random forest (RF), extreme gradient boosting (XGBoost), and k-nearest neighbor algorithm (KNN). The four models were evaluated by three aspects, i.e., discrimination, calibration, and clinical usefulness, and the best model was selected for prediction analysis. The ROC curve was plotted to obtain the AUC value and determine the predictive efficiency of the model; the calibration curve was plotted to assess whether there was good agreement between the predicted and actual results of the model; and decision curve analysis (DCA) was plotted to assess the benefit to the patient after interventional treatment. Internal validation was completed using the k-fold cross-validation method. (4) External validation of the best model using an external test set, plotting ROC curves and calibration curves, and determining the generalizability and predictive efficiency of the model. (5) Model interpretation. The contribution of each feature in the sample to the prediction is obtained by SHAP analysis, i.e., the Shapley value. The SHAP summary plot, which ranks the importance of risk factors, and the SHAP force plot, which analyzes and interprets the prediction results of individual samples, are constructed based on the Shapley values.

## 3. Results

### 3.1. Basic clinical information of the patient

**Figure 1:**
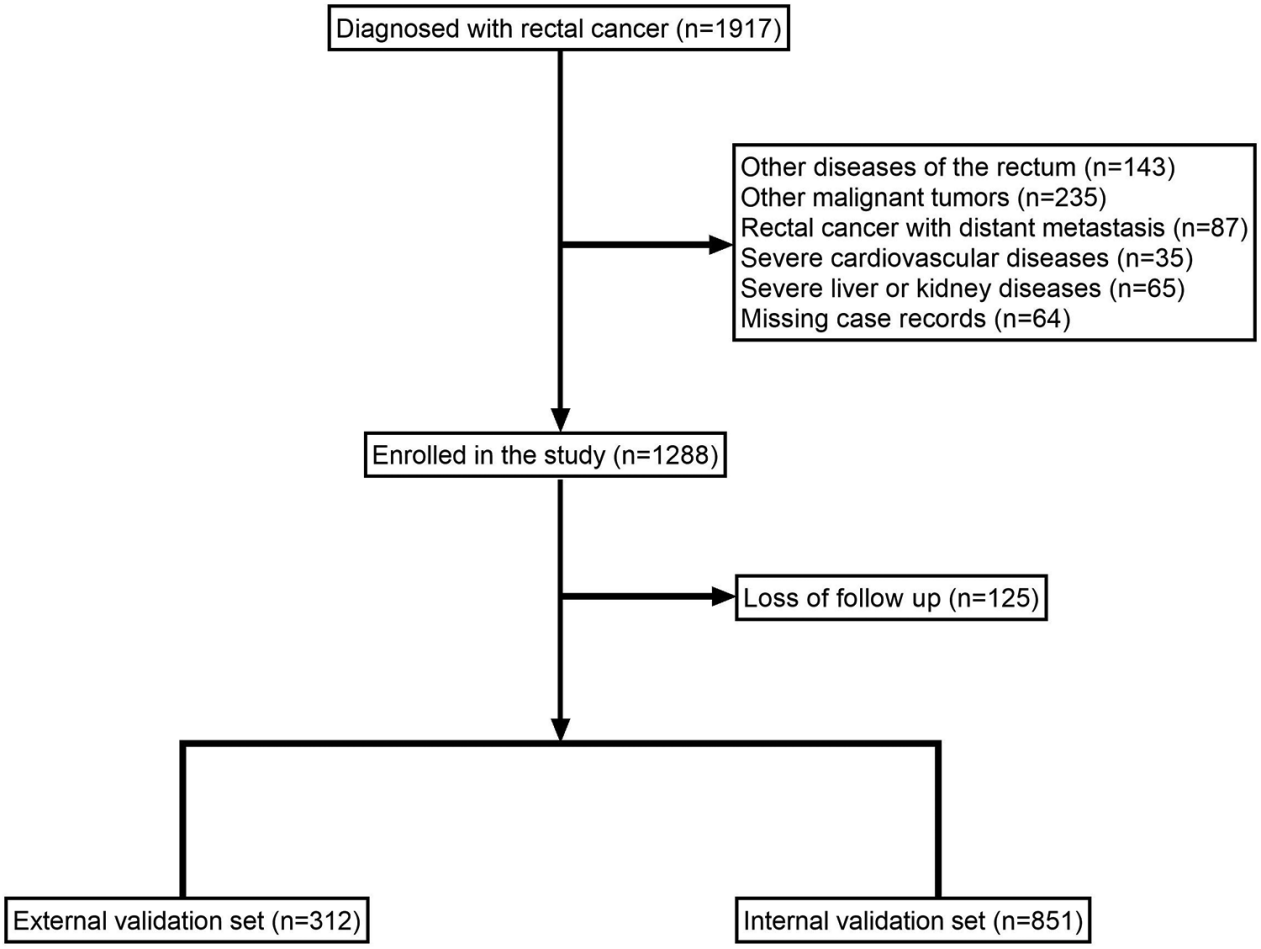
Flow diagram of patients included in the study.

A total of 1,163 patients were included in the study (Figure 1), including 142 (12.21%) patients with permanent stomas.

### 3.2. Analysis of risk factors for permanent stoma in patients with rectal cancer

The results of univariate analysis showed that there was a significant difference between the permanent stoma group and the nonpermanent stoma group in terms of age, history of hypertension, history of diabetes mellitus, tumor recurrence, history of adjuvant radiotherapy, history of adjuvant chemotherapy, distance of the tumor from the dentate line, and whether there was rectal stenosis (*P* < 0.05). The results of multivariate analysis showed that age ≥65 years, history of hypertension, history of diabetes mellitus, history of adjuvant radiotherapy, history of adjuvant chemotherapy, tumor distance ≥5 cm from the dentate line, and rectal stenosis were independent influencing factors for permanent stoma in patients with rectal cancer (*P* < 0.05) (Table 1).

**Table 1:** Univariate and multivariate analysis of variables related to permanent stoma.

Variants		Univariate analysis			Multivariate analysis		
		OR	95%CI	*P*-value	OR	95%CI	*P*-value
Sex	Female	Reference					
	Male	1.033	[0.693, 1.541]	0.873			
Age	<65	Reference			Reference		
	≥65	4.16	[2.290, 7.555]	<0.001	3.665	[1.44,10.434]	0.010
BMI	<25 kg/m^2^	Reference					
	≥25 kg/m^2^	1.24	[0.834, 1.843]	0.288			
ASA	<3	Reference					
	≥3	1.049	[0.707, 1.556]	0.811			
Drinking history	No	Reference					
	Yes	1.15	[0.773, 1.711]	0.490			
Smoking history	No	Reference					
	Yes	1.029	[0.694, 1.524]	0.888			
Surgical history	No	Reference					
	Yes	1.051	[0.704, 1.568]	0.808			
Anemia	No	Reference					
	Yes	1.044	[0.705, 1.548]	0.828			
Rectal stenosis	No	Reference			Reference		
	Yes	23.101	[14.240, 37.477]	<0.001	17.296	[8.201,39.173]	<0.001
Hyperlipidemia	No	Reference					
	Yes	1.347	[0.906, 2.003]	0.141			
Hypertensive	No	Reference			Reference		
	Yes	4.757	[3.121, 7.251]	<0.001	4.541	[2.188,9.783]	<0.001
Diabetes	No	Reference			Reference		
	Yes	3.858	[2.435, 6.114]	<0.001	4.316	[2.004,9.885]	<0.001
Coronary heart disease	No	Reference					
	Yes	0.954	[0.644, 1.414]	0.816			
T-category	T1∼T2	Reference					
	T3∼T4	1.069	[0.717, 1.595]	0.744			
N-category	N0	Reference					
	N1∼N2	0.921	[0.613, 1.384]	0.692			
Tumor size	<5 cm	Reference					
	≥5 cm	0.725	[0.479, 1.099]	0.130			
Tumour recurrence	No	Reference			Reference		
	Yes	3.05	[1.573, 5.911]	0.001	1.302	[0.412,3.997]	0.647
Distance from dentate line	≥5 cm	Reference			Reference		
	<5 cm	52.999	[31.188, 90.063]	<0.001	34.79	[16.223,80.558]	<0.001
Adjuvant Radiotherapy	No	Reference			Reference		
	Yes	2.817	[1.797, 4.417]	<0.001	2.652	[1.251,5.865]	0.013
Adjuvant Chemotherapy	No	Reference			Reference		
	Yes	12.084	[7.206, 20.266]	<0.001	8.816	[4.073,20.545]	<0.001
Albumin	<30 g/L	Reference					
	≥30 g/L	0.73	[0.490, 1.088]	0.122			
CEA level	<5 ng/ml	Reference					
	≥5 ng/ml	0.93	[0.627, 1.378]	0.716			
CA19-9 level	<37 U/ml	Reference					
	≥37 U/ml	0.843	[0.569, 1.250]	0.395			
NRS2002 score	<3	Reference					
	≥3	0.714	[0.456, 1.119]	0.142			

OR, odds ratio; CI, confidence interval; BMI, body mass index; ASA, The American Society of Anesthesiologists; ALB, albumin; CEA, carcinoembryonic antigen; CA19-9, carbohydrate antigen 19-9; NRS2002, nutrition risk screening 2002.

### 3.3. Model building and evaluation

**Figure 2:**
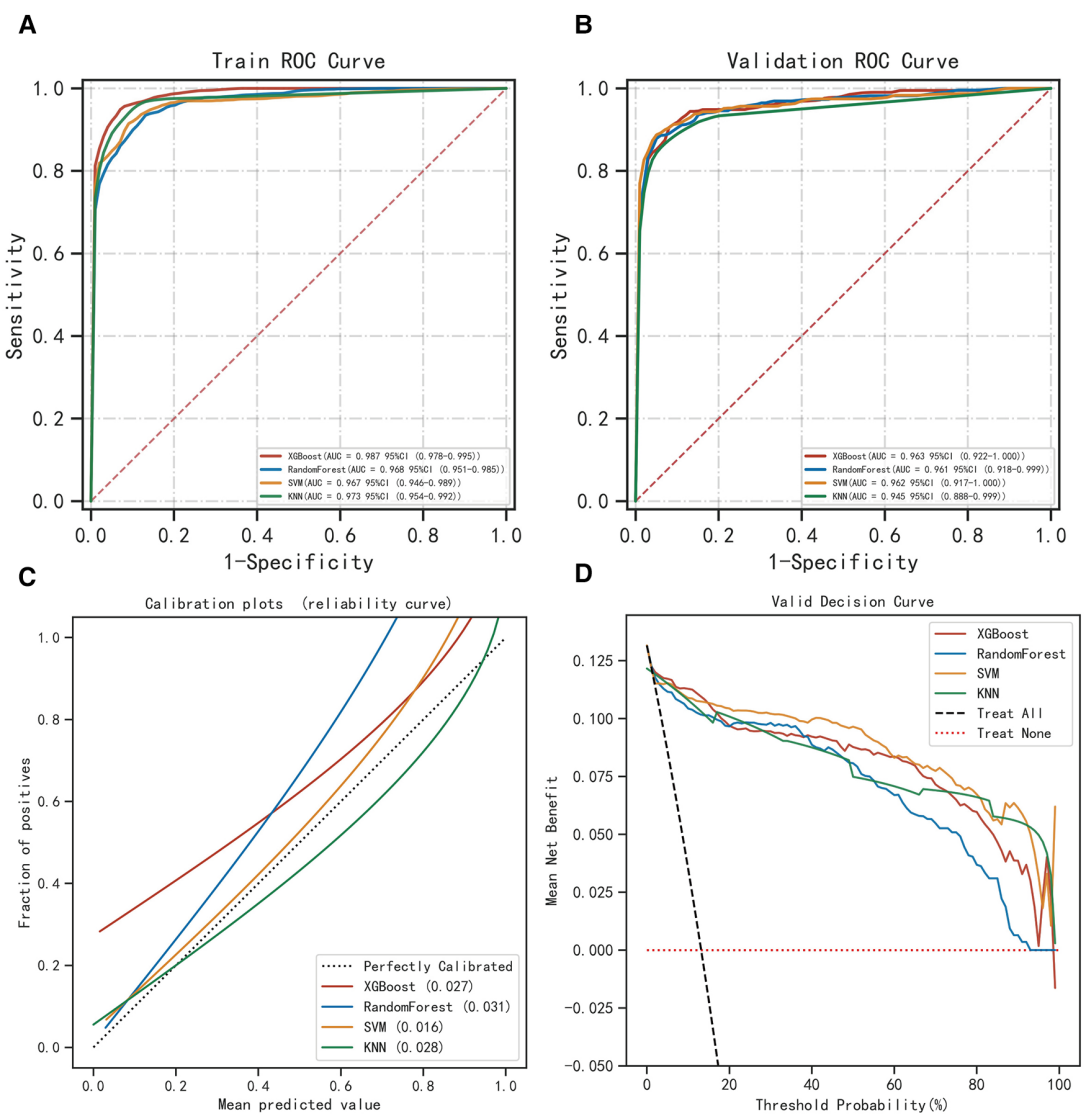
Evaluation of the four models for predicting permanent stoma. (**A**) ROC curves for the training set of the four models. (**B**) ROC curves for the validation set of the four models. (**C**) Calibration plots of the four models. The 45° dotted line on each graph represents the perfect match between the observed (*y*-axis) and predicted (*x*-axis) complication probabilitys. A closer distance between two curves indicates greater accuracy. (**D**) DCA curves of the four models. The intersection of the red curve and the All curve is the starting point, and the intersection of the red curve and the None curve is the node within which the corresponding patients can benefit.

**Figure 3:**
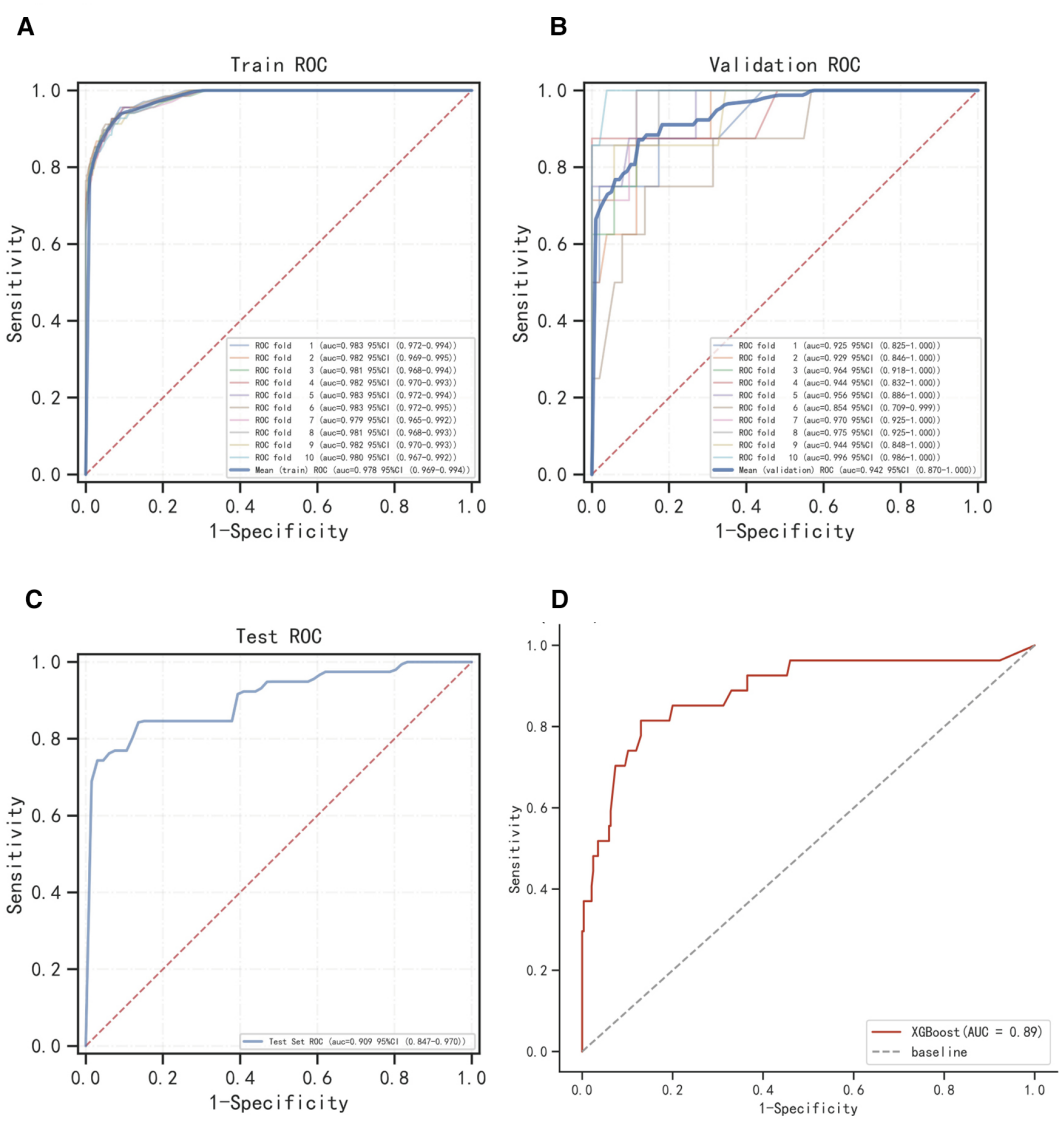
Internal validation of XGBoost model. (**A**) ROC curve of XGBoost model for the training set. (**B**) ROC curve of XGBoost model for the validation set. (**C**) ROC curve of XGBoost model for the test set. (**D**) External validation of XGBoost model.

The ROC curve results show that XGBoost has an AUC value as high as 0.987 in the training set; the AUC value in the validation set is 0.963, which is the best performance among the four models (Figures 2A,B, Table 2). The calibration curve results show that the calibration curves of the four models are similar to the ideal curves, and the models have high consistency between the predicted and actual results (Figure 2C). The DCA curves showed that all four models achieved a net clinical benefit relative to either the full treatment or no treatment plan (Figure 2D). The k-fold cross-validation method was used to compare the generalization ability of the four models. Taking the test set *N* = 256 cases (30.08%) and the remaining samples as the training set for 10-fold cross-validation, AUC = 0.9456 ± 0.0367 in the validation set of XGBoost, AUC = 0.9088 in the test set, accuracy = 0.9102 (Figures 3A–C); AUC = 0.9025 ± 0.0627 in the validation set of RF, AUC = 0.8789 in the test set, accuracy = 0.8750; AUC = 0.9274 ± 0.0440 in the validation set of SVM, AUC = 0.9006 in the test set, accuracy = 0.9414; AUC = 0.8720 ± 0.0713 in the validation set of KNN, AUC = 0.8743 in the test set, accuracy = 0.9336. After a comprehensive comparison, the XGBoost algorithm was chosen to construct the model in this study.

**Table 2:** Evaluation of the performance of the four models.

		AUC(95%CI)	Accuracy(95%CI)	Sensitivity(95%CI)	Specificity(95%CI)	F1 Score(95%CI)
KNN	Training set	0.973 (0.954–0.992)	0.954 (0.950-0.958)	0.946 (0.922-0.969)	0.910 (0.889-0.931)	0.883 (0.865-0.900)
	Validation set	0.945 (0.888–0.999)	0.949 (0.938–0.959)	0.879 (0.833–0.925)	0.931 (0.900–0.963)	0.838 (0.795–0.881)
XGBoost	Training set	0.987 (0.978–0.995)	0.934 (0.928–0.939)	0.957 (0.948–0.965)	0.927 (0.920–0.933)	0.799 (0.786–0.813)
	Validation set	0.963 (0.922–1.000)	0.916 (0.902–0.929)	0.926 (0.888–0.964)	0.923 (0.903–0.943)	0.743 (0.699–0.787)
RandomForest	Training set	0.968 (0.951–0.985)	0.897 (0.885–0.909)	0.925 (0.902–0.948)	0.890 (0.872–0.908)	0.714 (0.692–0.735)
	Validation set	0.961 (0.918–0.999)	0.889 (0.868–0.910)	0.890 (0.852–0.927)	0.961 (0.945–0.976)	0.678 (0.622–0.735)
SVM	Training set	0.967 (0.946–0.989)	0.911 (0.905–0.916)	0.923 (0.910–0.935)	0.906 (0.898–0.914)	0.740 (0.731–0.749)
	Validation set	0.962 (0.917–1.000)	0.897 (0.880–0.914)	0.909 (0.869–0.950)	0.951 (0.932–0.970)	0.697(0.644–0.750)

CI, confidence interval.

### 3.4. Model external validation

The results of the ROC curve showed that the AUC value of the external validation set was 0.89, indicating that the prediction model was highly accurate in determining the disease (Figure 3D).

### 3.5. Model explanation

**Figure 4:**
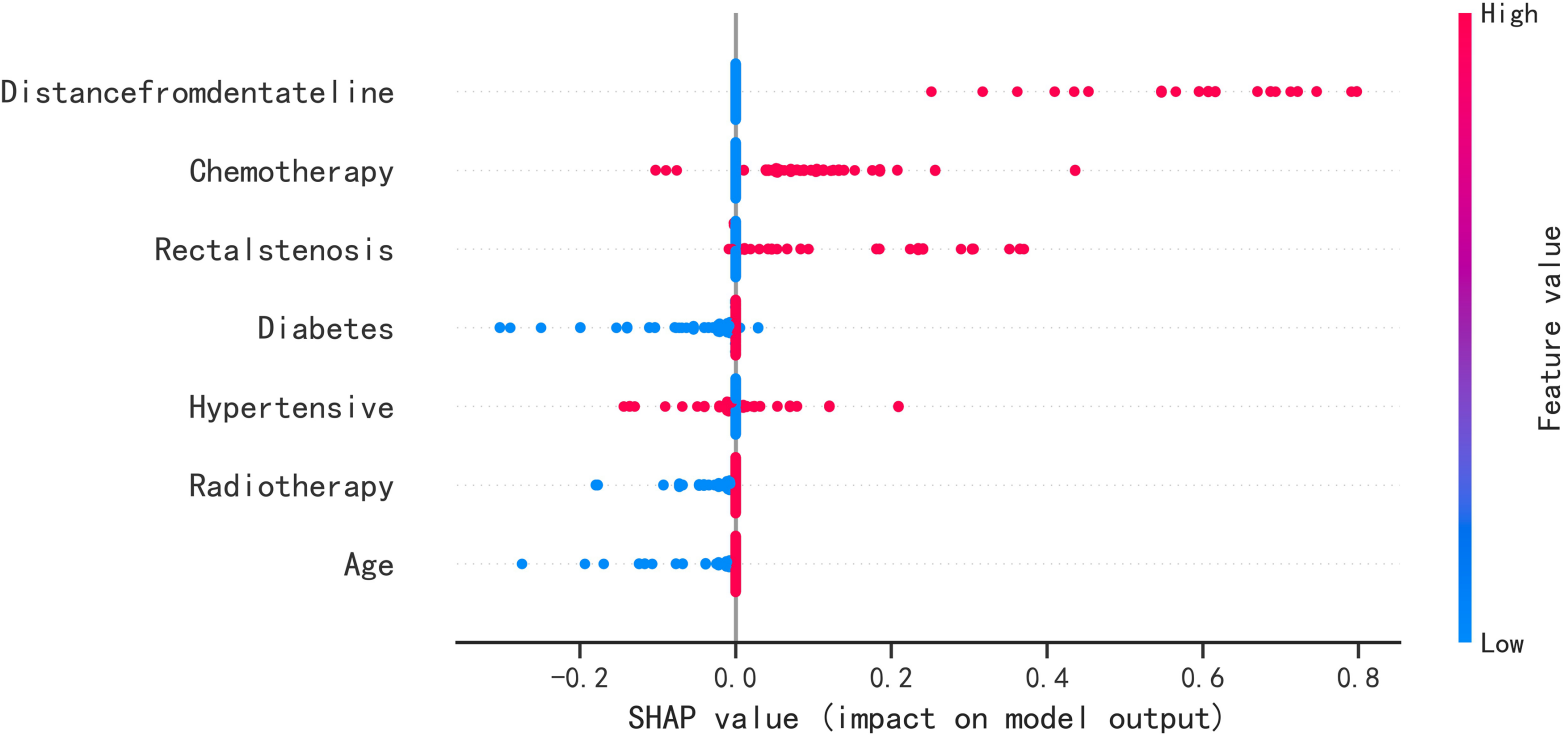
SHAP summary plot. Risk factors are arranged along the y-axis based on their importance, which is given by the mean of their absolute Shapley values. The higher the risk factor is positioned in the plot, the more important it is for the model.

**Figure 5:**
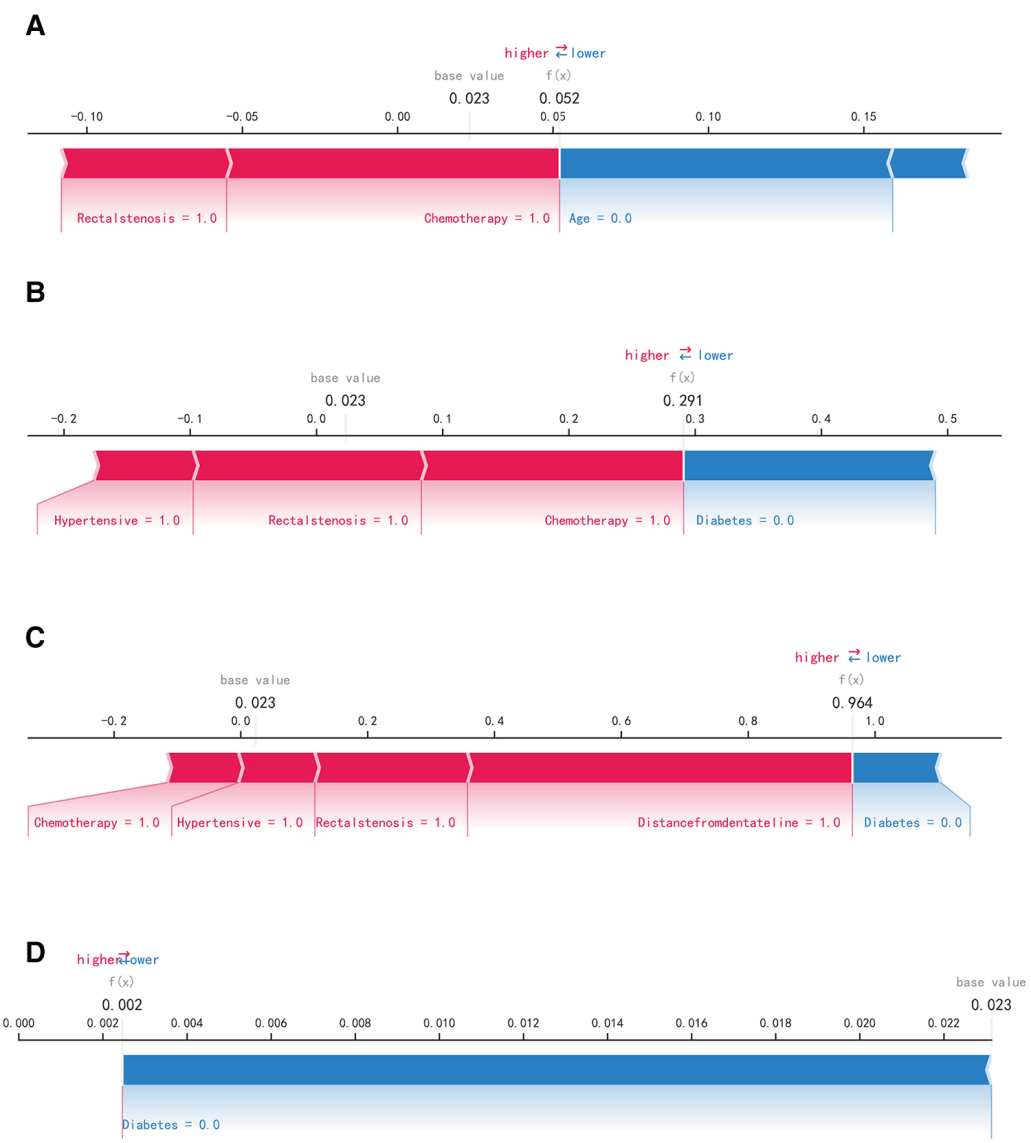
SHAP force plot. The contributing variables are arranged in the horizontal line, sorted by the absolute value of their impact. Blue represents features that have a negative effect on disease prediction, with a decrease in SHAP values; red represents features that have a positive effect on disease prediction, with an increase in SHAP values. (**A**) Predictive Analysis of Patient I. (**B**) Predictive Analysis of Patient II. (**C**) Predictive Analysis of Patient III. (**D**) Predictive Analysis of Patient IV.

The SHAP summary plot results showed that the risk factors for permanent stoma in patients with rectal cancer were ranked as tumor distance from the dentate line ≥5 cm, history of adjuvant chemotherapy, rectal stricture, history of diabetes mellitus, history of hypertension, history of adjuvant radiotherapy, and age ≥65 years (Figure 4). The SHAP force plot shows the predictive analysis of the study model for four patients with rectal cancer with permanent stoma. The model predicts a 0.052 probability of permanent stoma in patient I, with an increased probability of chemotherapy and rectal stenosis and a decreased probability of age <65 years; the model predicts a 0.291 probability of permanent stoma in patient II, with an increased probability of history of hypertension, chemotherapy, and rectal stenosis and a decreased probability of no history of diabetes; the model predicts a 0.964 probability of permanent stoma in patient III, and the probability was increased by history of chemotherapy, history of hypertension, rectal stenosis, and tumor <5 cm from the dentate line, and decreased by the patient's lack of diabetes; the model predicted a 0.002 probability of permanent stoma in patient IV, and decreased by the patient's lack of diabetes (Figures 5A–D).

## 4. Discussion

The current study evaluated risk prediction models constructed by four machine learning algorithms. Among them, the XGBoost algorithm exhibited the highest accuracy and was efficient, flexible, and universally adaptable (13). Compared with the RF algorithm, the XGBoost algorithm takes full account of the regularization problem and can effectively avoid model overfitting. The SVM algorithm and KNN algorithm have higher accuracy and can avoid the problem of overfitting well, but the stability of the two algorithms is poor when solving problems with multiple features and large samples (14). The XGBoost algorithm is more suitable for multidimensional studies and reduces the computation and training time. Compared with the SVM algorithm and KNN algorithm, the XGBoost algorithm is more advantageous. Therefore, with a comprehensive comparison of four machine learning algorithms, this study chose to use the XGBoost algorithm to construct a model to predict permanent stoma in patients with rectal cancer. Some studies (15, 16) have validated the effectiveness of machine learning algorithm applications in clinical diagnosis as well as prognosis. Moreover, machine learning techniques can also accurately predict adverse outcomes in disease progression compared to traditional diagnostic methods. Machine learning algorithms also played a great role in building the prediction model in this study. The model in this study can help clinical decision makers accurately identify high-risk patients, provide timely interventional treatment and improve patient prognosis. On the other hand, the model can help medical institutions allocate medical resources rationally, focus on the vital signs of high-risk patients, and effectively improve the survival rate of rectal cancer patients. Moreover, this study also used SHAP analysis to explain the model, and the results showed that advanced age, distance of the tumor from the dentate line, rectal stenosis, history of diabetes, history of hypertension, history of adjuvant chemotherapy, and history of adjuvant radiotherapy were risk factors for permanent stoma in patients with rectal cancer.

Patients of advanced age and those with a history of underlying medical conditions such as hypertension and diabetes mellitus are in poor physical condition. Patients have sclerotic and poorly dilated vascular walls that are friable (17–19). Additionally, their coagulation function is altered to some extent (20), which prevents rapid physiological hemostasis. All these factors lead to a weakened blood supply to the patient's gastrointestinal tract and a greater risk of postoperative anastomotic leakage. Some elderly patients do not have high quality of life requirements and have their own concerns about secondary surgery, so clinicians may prefer to consider permanent stoma for such patients. In addition, four samples were used in this study to explain how the model predicted permanent ostomy. For example, in the disease prediction analysis of samples I, II and III, a history of chemotherapy was one of the significant risk factors. Chemotherapy is often used as a treatment option for malignant tumors such as rectal cancer and liver cancer, and is highly selective to maximize inhibition of tumor growth and spread (21, 22). However, some of the chemotherapy modalities are more irritating to the abdominal cavity and aggravate the degree of abdominal adhesions. It also inhibits the normal physiological function of the bone marrow, and patients experience postoperative anemia and immune dysfunction, which affects the near and long-term outcome. On the other hand, patients can have severe abdominal inflammatory reactions as well as gastrointestinal reactions, which do not prevent the occurrence of intestinal obstruction (23). Studies by Makrin et al. (24, 25) also demonstrated that intraperitoneal chemotherapy has a detrimental effect on the recovery of the gastrointestinal tract and is most likely one of the risk factors for postoperative intestinal obstruction, further confirming the higher risk of permanent stoma in patients treated with chemotherapy. The current study also found that radiotherapy is one of the risk factors for the permanence of stoma in patients. Patients with rectal cancer choose radiotherapy to reduce the postoperative recurrence rate of the tumor and to improve survival. Zhu et al. (26, 27) discussed the effectiveness of postoperative radiotherapy for rectal cancer and demonstrated that this treatment modality can significantly reduce the local recurrence rate of rectal cancer and improve the quality of survival of patients. However, as with intraperitoneal chemotherapy, radiation therapy is more damaging to the gastrointestinal tract. On the one hand, it restricts its peristaltic function and causes stiffness of the intestinal wall, which in the long run decreases the compliance of the intestine; on the other hand, radiotherapy directly damages intestinal epithelial cells and vascular endothelial cells, and the intestinal wall gradually fibroses. Some studies (28) performed pathological biopsies of tumors in most patients treated with adjuvant radiotherapy and found reduced microvascular counts at the tumor cut edges, an increased percentage of stenotic vessels, and significant fibrosis of the surrounding intestinal wall. Additionally, a study by Kumagai et al. (29) showed that radiotherapy is highly susceptible to complications of intestinal perforation and intestinal obstruction, and these complications increase the risk of permanent stoma to some extent. The results of the current study also revealed a greater risk of perpetuation of the diseased artificial orifice in rectal stenosis. TME carries out a radical tumor resection with complete removal of the mesentery around the rectal cancer, which requires the operator to ligate at the beginning of the inferior mesenteric artery, which is highly likely to cause the intestinal canal at the anastomosis to be in a hypoxic and hypoperfused state. If patients with rectal stenosis remain in this state for a long time, the fibrous tissue of the intestinal canal will further proliferate, leading to severe postoperative bowel obstruction (27). Such patients not only feel severe pain at the anus during defecation but are also unable to undergo ostomy reversal, which further aggravates physical and psychological trauma. This suggests that clinicians should closely monitor the patient's vital status before surgery, prepare the intestine and prevent the occurrence of stenosis; select a reasonable surgical approach during surgery, fully free the colon to the splenic area to reduce anastomotic tension and ensure a good blood supply to the anastomosis; and perform regular postoperative rectal finger examination to dilate the anus and loosen the stenotic ring. Doctors can use balloon dilation to reduce the degree of stenosis when dealing with patients with mild stenosis symptoms; when dealing with patients with more severe stenosis symptoms, they should promptly inform patients of their condition, communicate well with doctors and patients, and reduce the psychological burden of patients. In addition, Mak (21) considered local recurrence of tumors as a risk factor for stoma permanence. His study showed that recurrent tumors occupy the intestinal space, making it more difficult for food to pass normally through the intestinal lumen, which can easily cause intestinal obstruction. Moreover, tumor infiltration of the intestinal canal causes stiffness of the intestinal wall, which weakens peristalsis and aggravates the degree of obstruction, so patients are highly susceptible to permanent stoma (21, 30, 31). However, in the present study, tumor recurrence was not a risk factor for permanent stoma in patients with rectal cancer. Our analysis suggests that this may be related to the small number of cases of rectal cancer recurrence in this study, and more relevant cases will be added in the future to improve the study. It also suggests that clinicians should strengthen the postoperative follow-up of rectal cancer patients, promptly review them after discovering discomfort, and intervene early to relieve symptoms.

The current study comprehensively evaluated the model in terms of discrimination, calibration, and clinical utility, but the study has some limitations. The study included multiple aspects of risk factors but did not consider aspects such as imaging. Although the machine learning algorithms were more accurate, their models were more complex and less interpretable. The entire computational and decision-making process of the model runs in a black box, which is not as intuitive and clear as the logistic regression model (32–34). On the other hand, the current study was a single-center retrospective study, which has the disadvantages of selection bias, distribution bias, and retrospective bias. It is necessary to add multicenter prospective studies to future studies to further increase the reliability of the results.

## 5. Conclusion

This study developed a model based on the XGBoost machine learning algorithm to predict the risk of permanent stoma in rectal cancer. The model has good prediction accuracy and clinical utility, which facilitates surgeons in diagnosing patients in a timely manner. The model predicted patients at high risk for permanent stoma and identified advanced age, distance of the tumor from the dentate line, rectal stenosis, history of diabetes, history of hypertension, history of chemotherapy, and history of radiotherapy as high risk factors.

## Data Availability

The original contributions presented in the study are included in the article/Supplementary Material, further inquiries can be directed to the corresponding author/s.
